# Supplementary figures and images for: Joint contribution of adaptation and neuronal population recruitment to response level in visual area MT: a computational model
Source: Sci Rep. 2025 Jul 10;15:24964. doi: 10.1038/s41598-025-07699-8 (PMC12246228; doi:10.1038/s41598-025-07699-8)

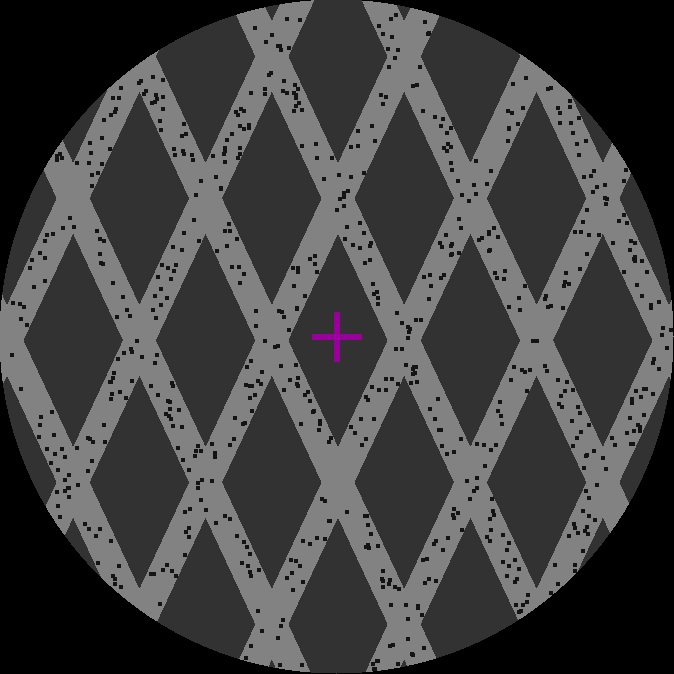

Supplement: Supplementary file 1 — Supplementary Material 1 [file 41598_2025_7699_MOESM1_ESM.zip › stimuli/S1 Coherent Stimulus.gif]

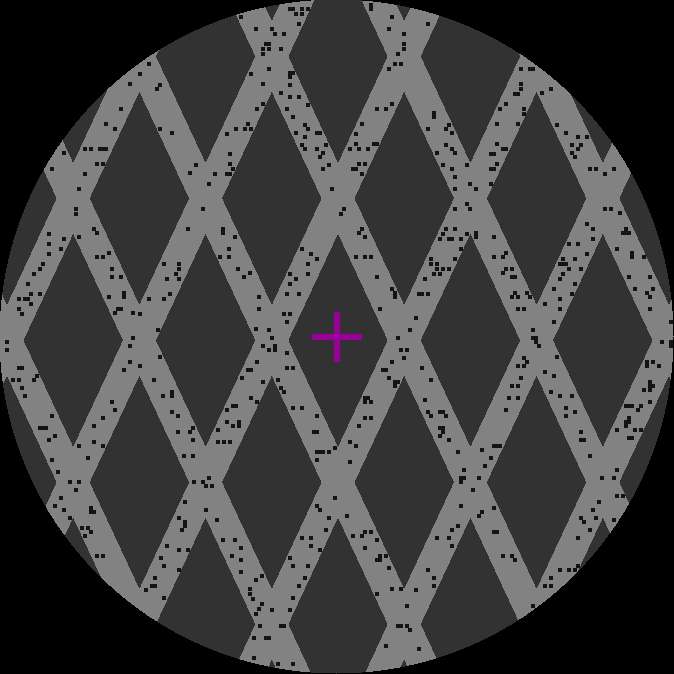

Supplement: Supplementary file 1 — Supplementary Material 1 [file 41598_2025_7699_MOESM1_ESM.zip › stimuli/S3 Non-Adapting Condition.gif]

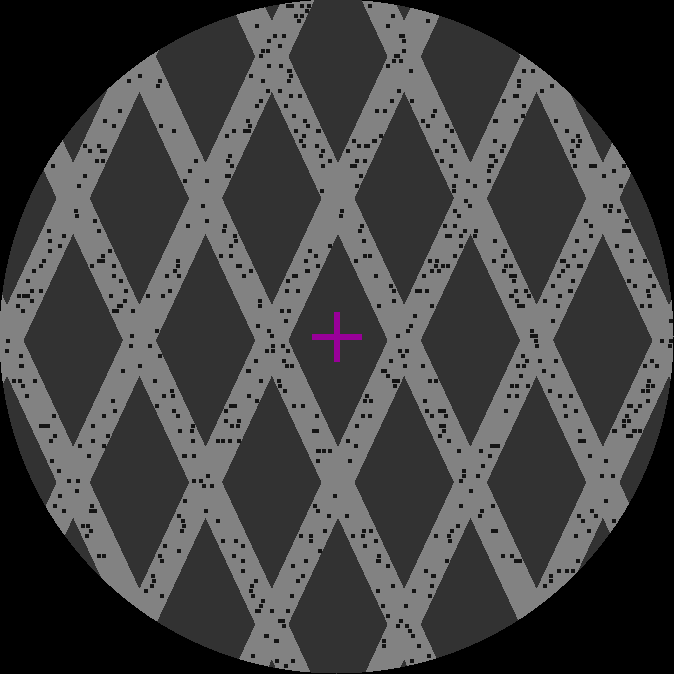

Supplement: Supplementary file 1 — Supplementary Material 1 [file 41598_2025_7699_MOESM1_ESM.zip › stimuli/S2 Incoherent Stimulus.gif]
